# Supplementary material for: Goal-Directed Fluid Therapy Enhances Gastrointestinal Recovery after Laparoscopic Surgery: A Systematic Review and Meta-Analysis
Source: J Pers Med. 2022 Apr 30;12(5):734. doi: 10.3390/jpm12050734 (PMC9143059; doi:10.3390/jpm12050734)
Supplement: Supplementary file 1 [file jpm-12-00734-s001.zip › jpm-1696074-supplementary.pdf]

Goal-directed fluid therapy enhances gastrointestinal recovery after laparoscopic surgery: a systematic review and meta-analysis

Marcell Virág[1,2,3], Máté Rottler[1,2,3], Noémi Gede[1], Klementina Ocskay[1,7], Tamás Leiner[1,4], Máté Tuba[1], Szabolcs Ábrahám[1], Nelli Farkas[1], Péter Hegyi[1,7,8] and Zsolt Molnár[1,3,5,6,7,*]

[1]Institute for Translational Medicine, Medical School, Szentágothai Research Centre, University of Pécs, Pécs, Hungary

[2]Department of Anesthesiology and Intensive Therapy, Szent György University Teaching Hospital of Fejér County, 8000 Székesfehérvár, Hungary

[3]Doctoral School of Clinical Medicine, University of Szeged, 6720 Szeged, Hungary

[4]Anaesthetic Department, Hinchingbrooke Hospital, North West Anglia NHS Foundation Trust, Huntingdon PE29 6NT, UK

[5]Department of Anaesthesiology and Intensive Therapy, Poznan University of Medical Sciences, 61-701 Poznan, Poland

[6]Department of Anaesthesiology and Intensive Therapy, Semmelweis University, 1082 Budapest, Hungary

[7]Centre for Translational Medicine, Semmelweis University, 1085 Budapest, Hungary

[8]Division for Pancreatic Disorders, Heart and Vascular Center, Semmelweis University, Budapest, Hungary

Correspondence to: Zsolt Molnár; zsoltmolna@gmail.com; Tel: +36-30-302-6668

Supplementary Table S1: List of accepted haemodynamic measurement as goal-directed fluid therapy 3
Supplementary Table S2: Detailed search 4
Supplementary Table S3: Baseline characteristics of the patients and length of operation of the included studies 5
Figure S1: Operation time 7
Figure S2: Funnel plot – Length of hospital stay 8
Figure S3: Funnel plot – Time to first stool 9
Figure S4: Funnel plot – Time to first flatus 10
Figure S5: Funnel plot – Intraoperative fluid requirement 11
Figure S6: Funnel plot – Intraoperative vasopressor requirement 12
Figure S7: Funnel plot – Intraoperative urinary output standardized for the length of the surgery 13
Figure S8: RoB – Length of hospital stay 14
Figures S9 RoB – Reoperation and readmission rate 15
Figures S10: RoB – Overall complication 16
Figure S11: RoB – Time to first flatus 17
Figure S12: RoB – Time to first stool 18
Figure S13: RoB – Intraoperative fluid requirement 19
Figure S14: RoB – Intraoperative vasopressor requirement 20
Figure S15: RoB – Intraoperative urinary output 21
Figure S16: RoB – Serum lactate levels at the end of the operation 22
Supplementary Table S4: Certainty assessment 23
Figure S17: Leave-one-out analysis – Length of hospital stay 24
Figure S18: Leave-one-out analysis – Time to first stool 25
Figure S19: Leave-one-out analysis – Time to first flatus 26
Figure S20: Leave-one-out analysis – Intraoperative fluid requirement 27
Figure S21: Leave-one-out analysis – Intraoperative vasopressor requirement 28
Figure S22: Leave-one-out analysis – Intraoperative urinary output 29

Supplementary Table S1: List of accepted haemodynamic measurement as goal-directed fluid therapy.

Technology	Measurements Derived	Name of Manufacturer(s) or Brand(s)
Transpulmonary indicator dilution	Cardiac output	LiDCO and PiCCO©
Arterial waveform-derived	Stroke volume variation and cardiac output	FloTrac™. LiDCO and PiCCO©
Oesophageal Doppler	Flow corrected time and stroke volume	CardioQ™
Partial CO2 rebreathing	Cardiac output	NICO©
Bioreactance	Cardiac output	NICOM©
Pulse oximetry	Pleth Variability Index	aMasimoSet

Supplementary Table S1: List of accepted haemodynamic measurement as goal-directed fluid therapy.

LiDCO: lithium dilution cardiac output (LiDCO Ltd. Lake Villa. Illinois. USA). NICO©: noninvasive cardiac output (Novametrix Medical Systems. Wallingford. Connecticut. USA). NICOM©: noninvasive cardiac output monitor (Cheetah Medical. Vancouver.Washington. USA). PiCCO©: pulse contour cardiac output (PULSION Medical Systems SE. Feldkirchen. Germany). FloTracTM (Edwards Life Sciences. Irvine. California. USA). CardioQ™ (Deltex Medical. Chichester. United Kingdom). aMasimo Set version V7.1.1.5 pulse oximeter (Masimo Co. Irvine. CA).

Supplementary Table S2: Detailed search.

Name of the Database	Date of Last Search	Field of Search	Restriction	Search Key	No of Records
Medline via PubMed®	26.10.2020	All fields	No restriction	(surgery OR laparoscop* OR peritoneoscop* OR intraoperative OR Perioperative OR Peri-operative) **AND** (fluid* OR "plasma substitute" OR "plasma volume" OR rehydration OR rehidration) **AND** (goal-directed OR goaldirected OR "haemodynamic monitor*" OR "hemodynamic monitor*" OR "hemodinamic monitor*" OR "perioperative monitor*" OR "stroke volume index" OR SVI OR "stroke volume varia*" OR SVV OR "cardiac index" OR "arterial pulse" OR "arterial pressure" OR vigil OR flotrac OR proAQT OR "Lithium Dilution Cardiac Output" OR LIDCO OR "noninvasive cardiac output monitor*" OR nicom OR "oesophageal doppler") **AND** random*	890
Embase®		All fields			1609
CENTRAL*		All fields			959
Web of Science		All fields			1088
SCOPUS®		Title, abstract, keywords			938

Supplementary Table S2: Detailed search.

* Cochrane Central Register of Controlled Trials.

Supplementary Table S3: Baseline characteristics of the patients and length of operation of the included studies.

Author	GDFT	N-GDFT	Age (Mean/SD)		Sex (M/F)		ASA I (No. of patients)		ASA II (No. of patients)		ASA III (No. of patients)		ASA IV (No. of patients)		Operation time (min) Mean; (SD)	
			GDFT	NGDFT	GDFT	NGDFT	GDFT	NGDFT	GDFT	NGDFT	GDFT	NGDFT	GDFT	NGDFT	GDFT	NGDFT
Gomez-Izquierdo 2017	68	64	63 (15)	61 (15)	31/33	40/24	6	8	42	38	14	18	2	0	220.0 (94.8)	210.0 (60.0)
Demirel 2017	30	30	40 (11.9)	36.3 (10.8)	11/19	12/18	0	0	15	12	15	18	0	0	98.0 (34.7)	109.0 (33.2)
Joosten 2018	20	20	Not reported		7/13	4/15	6	4	13	13	1	2	0	0	149.0 (62.2)	160.7 (134.6)
Liu 2019	37	37	69.2 (4.8)	70.3 (4.8)	23/14	23/14	6	5	31	32	0	0	0	0	185.1 (22.4)	177.6 (22.9)
Mei 2018	60	60	51 (10)	49.4 (9.5)	25/33	27/31	32	33	26	25	0	0	0	0	233.4 (41.1)	229.8 (41.7)
Ratti 2016	45	45	58 (11)	61 (9)	24/21	24/21	8	6	32	35	5	4	0	0	220.0 (50.0)	210.0 (60.0)
Wen 2016	40	40	Not reported													
Yin 2018	22	23	69.4 (6.4)	68.3 (5.8)	11/11	13/10	0	0	11	12	14	13	0	0	204.0 (36.0)	198.0 (42)
Li 2021	30	30	56.5 (5.0)	54.5 (5.0)	0/30	0/30	13	14	17	16	0	0	0	0	180.0 (13.0)	184.0 (12.0)
Mühlbacher 2021	30	30	38.0 (11.0)	39.0 (11.0)	6/24	5/25	3	4	24	19	3	7	0	0	110.0 (33.0)	106.0 (41.0)

Tang 2021	37	37	N/A	N/A	24/13	24/13	7	5	16	21	14	11	0	0	N/A	N/A

Supplementary Table S3: Baseline characteristics of the patients and length of operation of the included studies.

GDFT: goal-direct fluid therapy; N-GDFT: non-goal-directed fluid therapy; SD: standard deviation; M/F: male/female; ASA: American Society of Anaestesiologists physical status classification system; N/A: data not available.

Figure S1: Operation time.

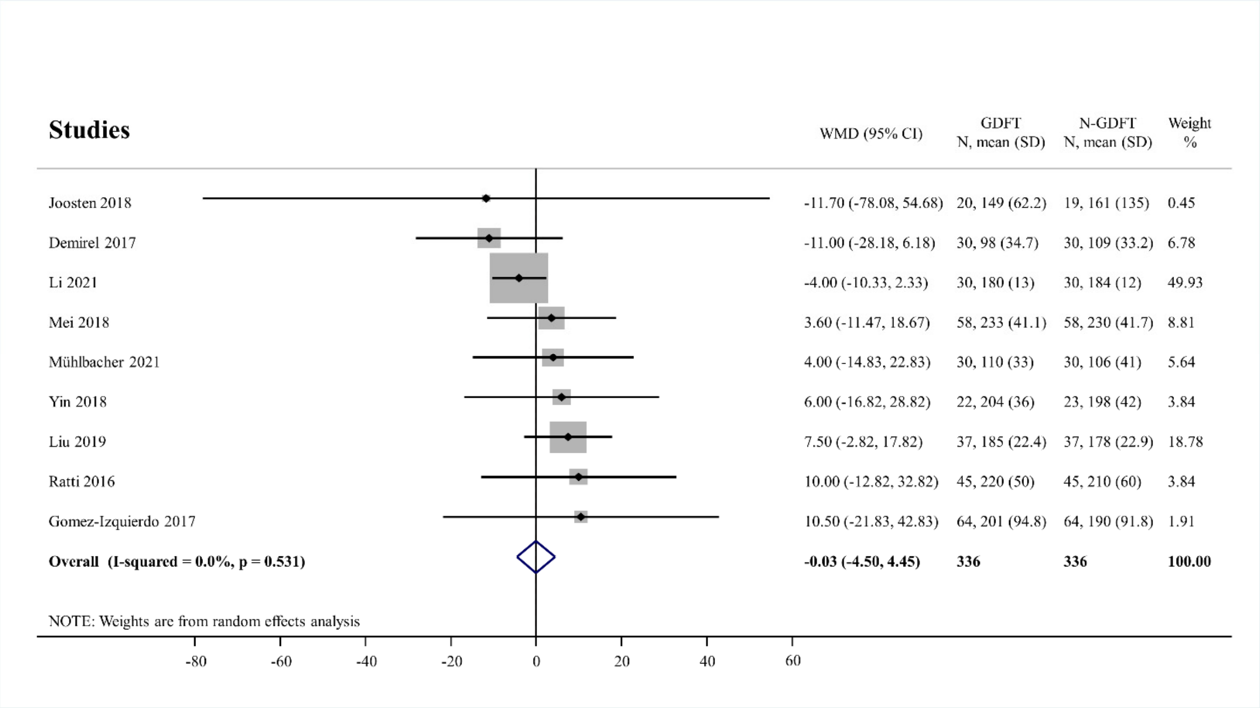

Supplementary Figure S1: Operation time (minutes)—forest plot.

WMD: weighted mean difference; SD: standard deviation; GDFT: goal-directed fluid therapy; N-GDFT: non-goal-directed fluid therapy; CI: confidence interval. P < 0.1 was considered significant.

Figure S2: Funnel plot—Length of hospital stay.

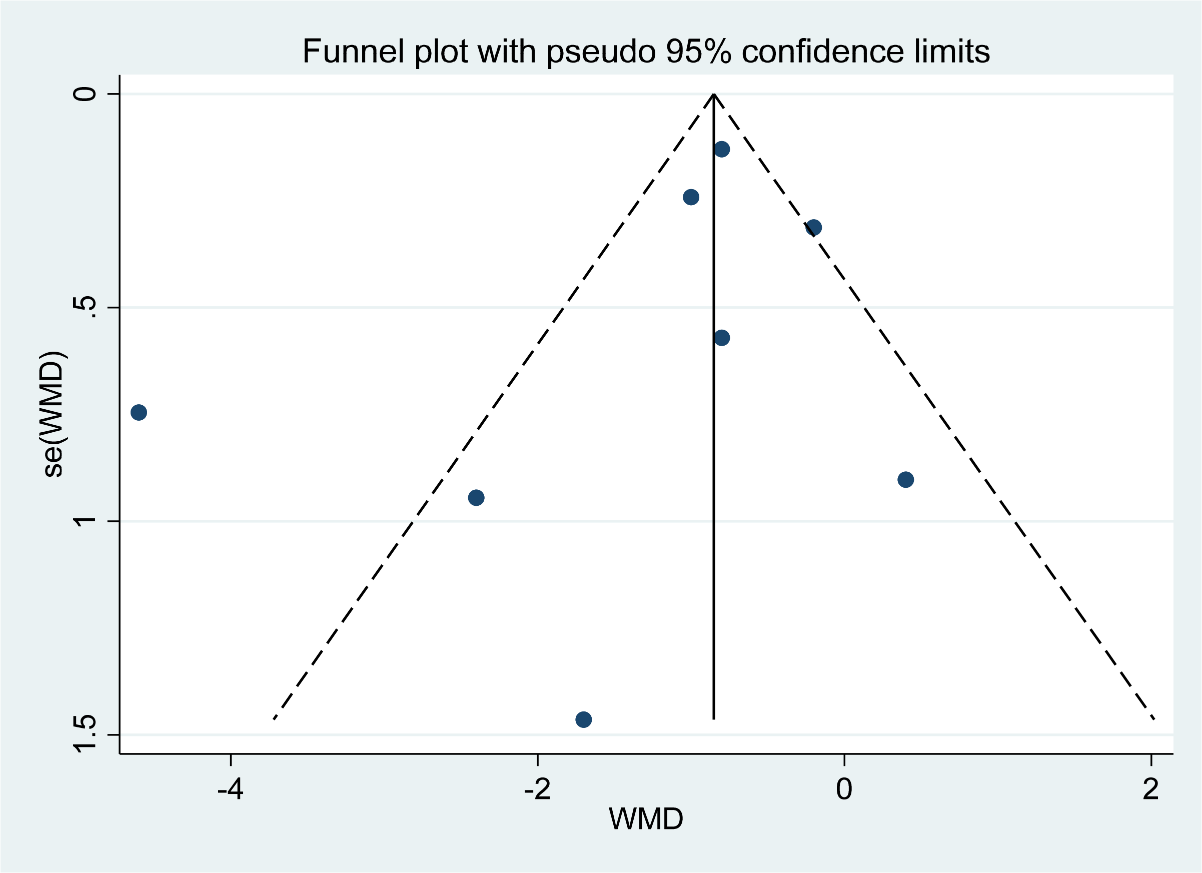

Supplementary Figure S2: Funnel plot—Length of hospital stay.

Funnel plot of studies that were included in comparison for length of hospital stay (days) between goal and non-goal-directed fluid therapy. The dots represent the results of each study included in the forest plot. WMD: weighted mean difference; se(WMD): standard error of weighted mean difference.

Figure S3: Funnel plot—Time to first stool.

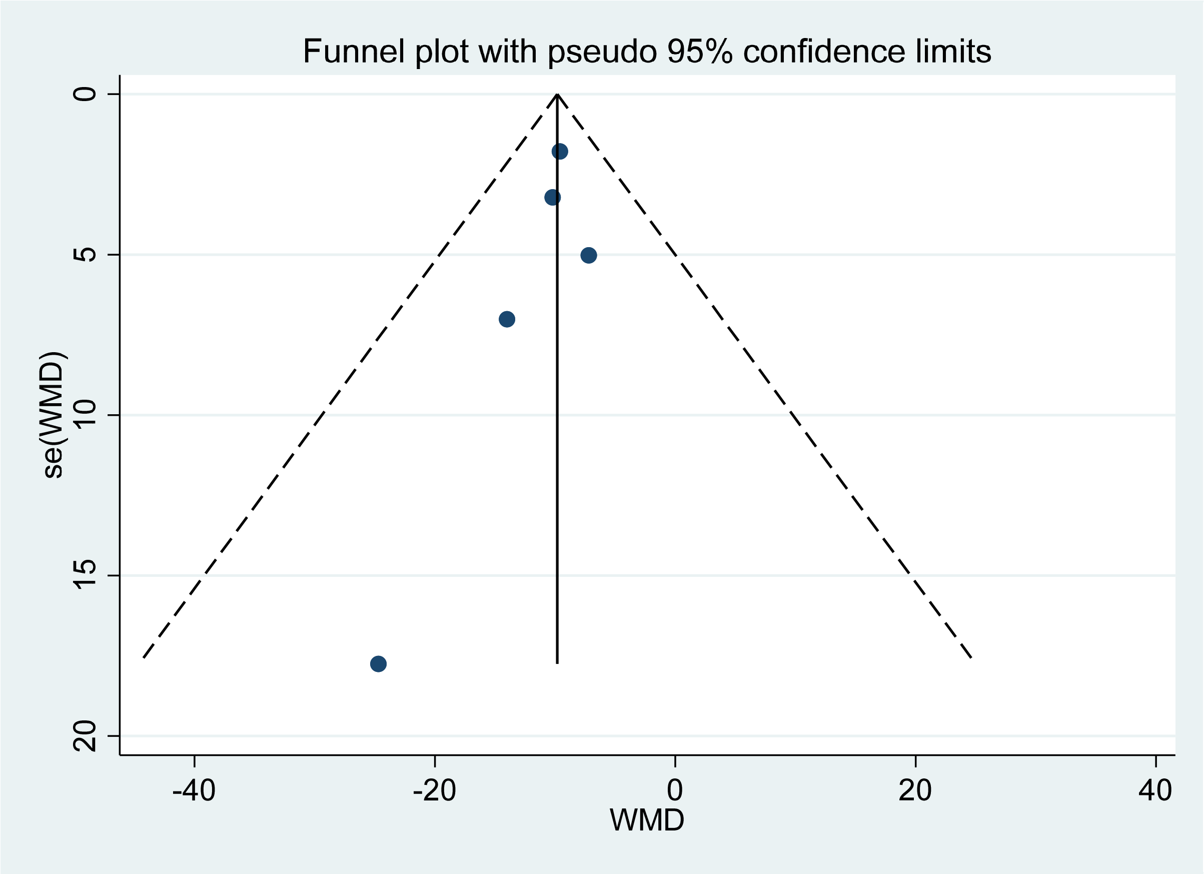

Supplementary Figure S3: Funnel plot—Time to first stool.

Funnel plot of studies that were included in comparison for time to first stool (hours) between goal- and non-goal-directed fluid therapy. The dots represent the results of each study included in the forest plot. WMD: weighted mean difference; se(WMD): standard error of weighted mean difference.

Figure S4: Funnel plot—Time to first flatus.

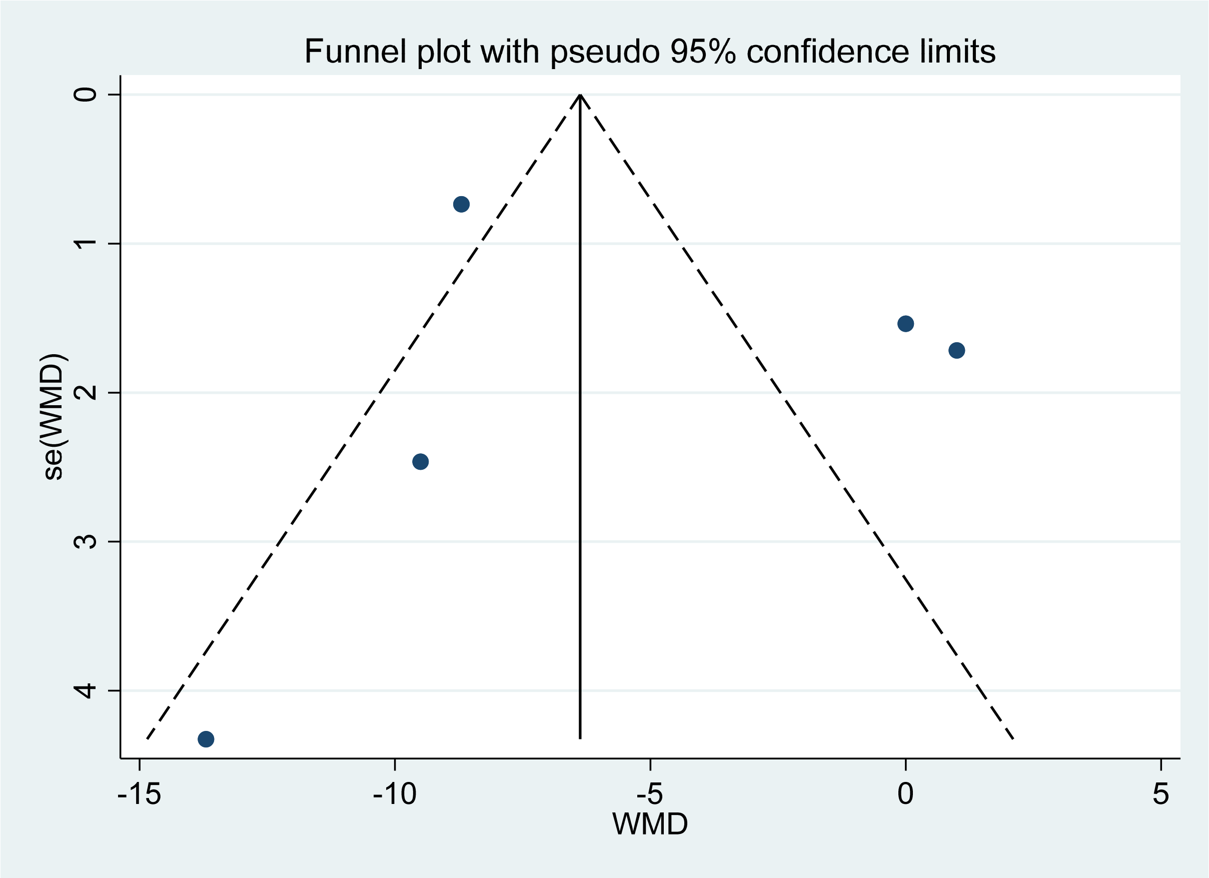

Supplementary Figure S4: Funnel plot—Time to first flatus.

Funnel plot of studies that were included in comparison for time to first flatus (hours) between goal- and non-goal-directed fluid therapy. The dots represent the results of each study included in the forest plot. WMD: weighted mean difference; se(WMD): standard error of weighted mean difference.

Figure S5: Funnel plot—Intraoperative fluid requirement.

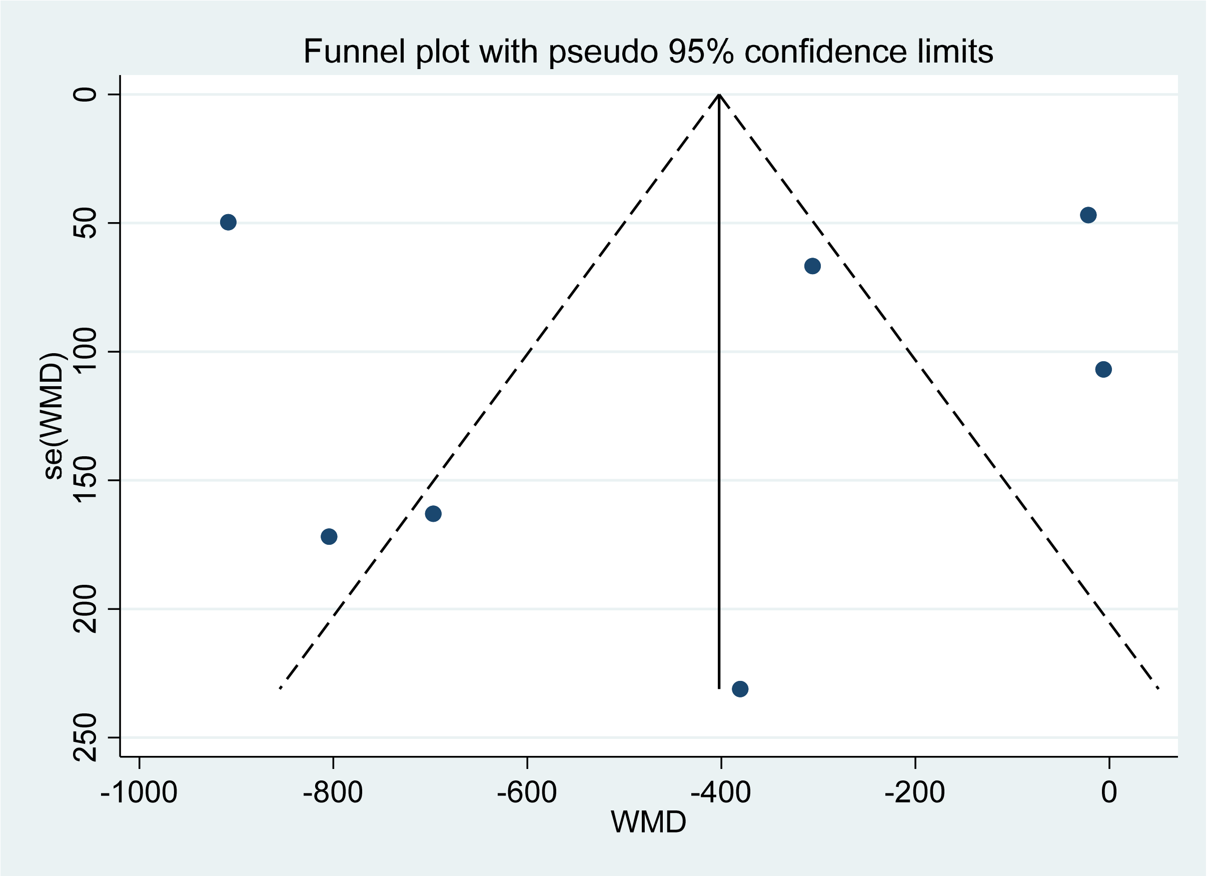

Supplementary Figure S5: Funnel plot—Intraoperative fluid requirement.

Funnel plot of studies that were included in comparison for intraoperative fluid requirement (mL) between goal- and non-goal-directed fluid therapy. The dots represent the results of each study included in the forest plot. WMD: weighted mean difference; se(WMD): standard error of weighted mean difference.

Figure S6: Funnel plot—Intraoperative vasopressor requirement.

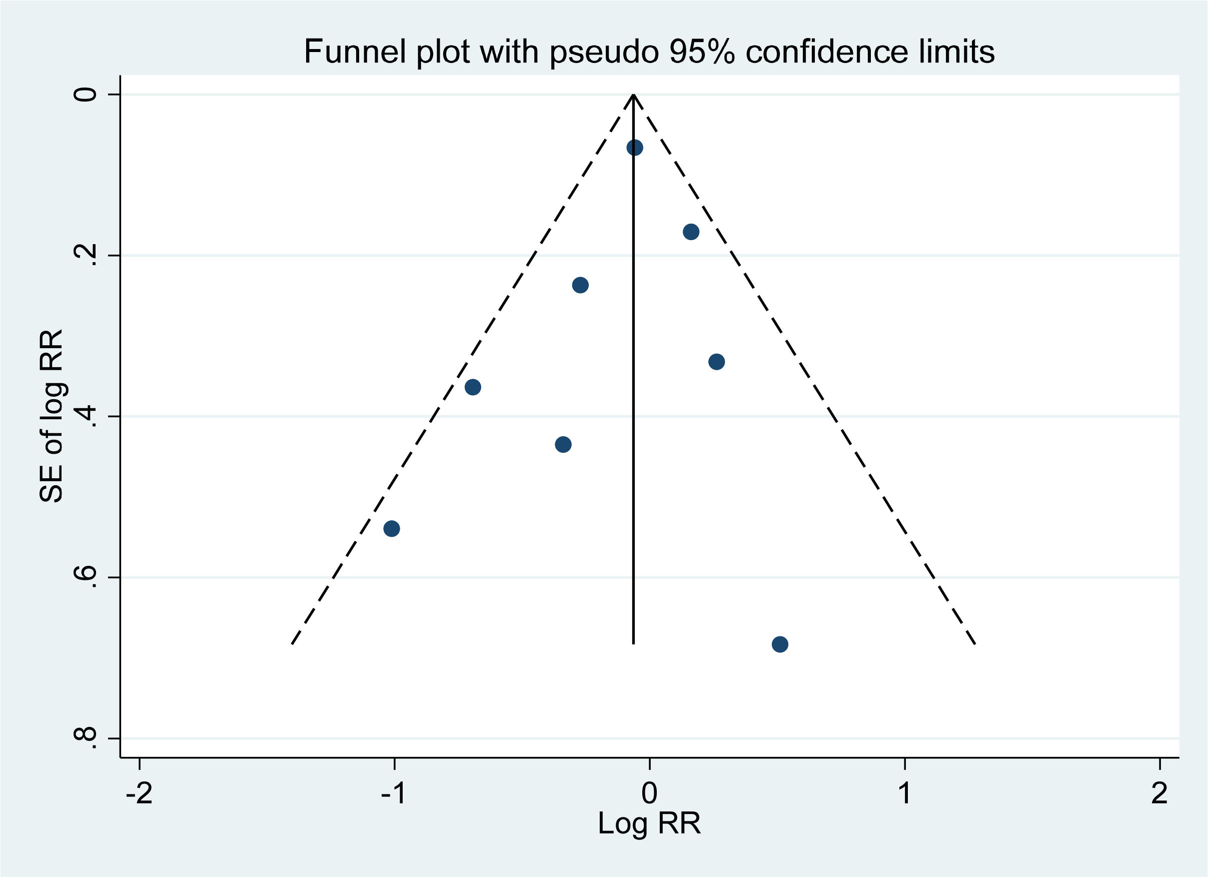

Supplementary Figure S6: Funnel plot—Intraoperative vasopressor requirement.

Funnel plot of studies that were included in comparison for intraoperative vasopressor requirement (%) between goal- and non-goal-directed fluid therapy. The dots represent the results of each study included in the forest plot. Log RR: logarithmic risk ratio; SE of (log RR): standard error of logarithmic risk ratio.

Figure S7: Funnel plot—Intraoperative urinary output standardized for the length of the surgery.

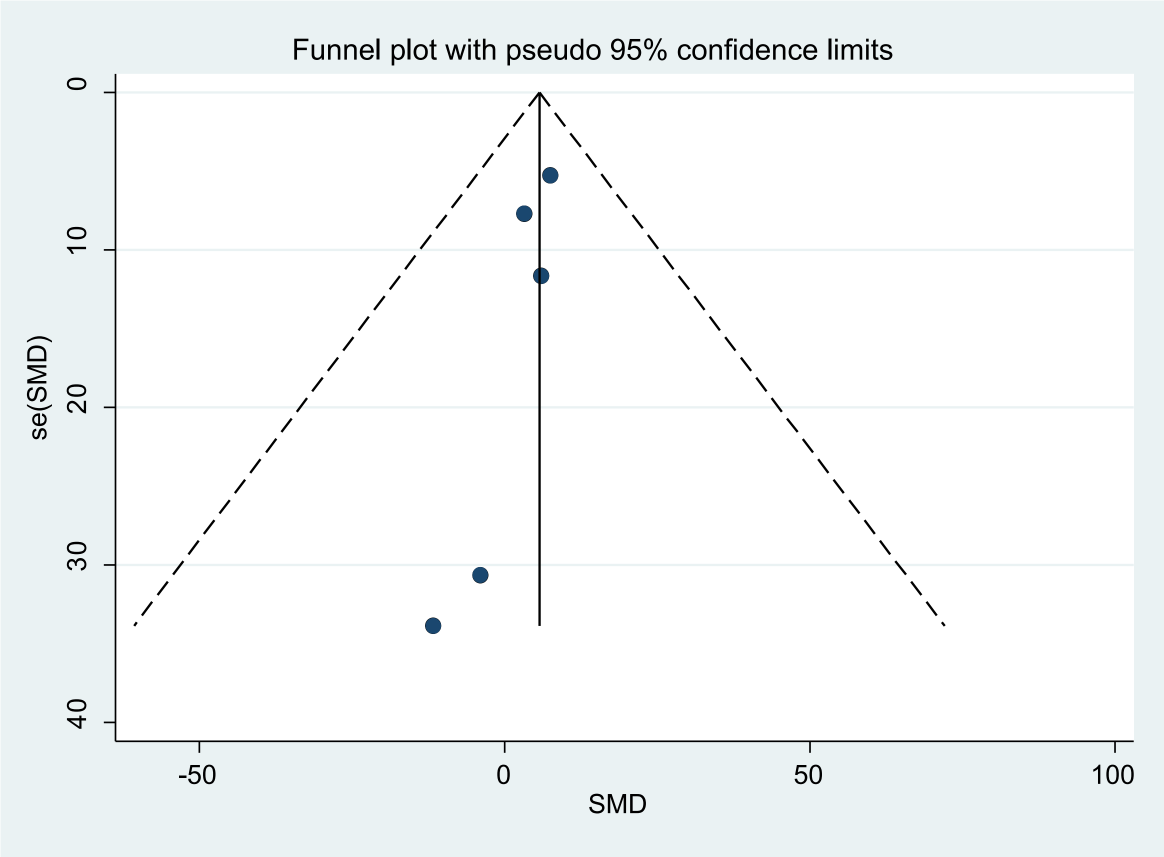

Supplementary Figure S7: Funnel plot—Intraoperative urinary output standardized for the length of the surgery.

Funnel plot of studies that were included in comparison for intraoperative urinary output standardized for the length of the surgery (mL h^{-1}) between goal- and non-goal-directed fluid therapy. The dots represent the results of each studies included in the forest plot. SMD: standardized mean difference; se(SMD): standard error of standardized mean difference.

Figure S8: RoB—Length of hospital stay.

A

+ Low risk
? Some concerns
– High risk

	Randomization process	Deviations from intended interventions	Missing outcome data	Measurement of the outcome	Selection of the reported result	**Overall**
Ratti, 2016	+	+	+	+	?	**?**
Gomez-Izquierdo, 2017	+	+	+	+	+	**+**
Yin, 2018	+	+	+	?	+	**?**
Mei, 2018	?	?	+	+	?	**?**
Liu, 2019	+	+	+	+	?	**?**
Joosten, 2018	+	+	+	+	+	**+**
Tang, 2021	+	+	+	+	?	**?**
Cho, 2021	+	+	+	+	?	**?**
Li, 2021	+	+	+	+	?	**?**

B

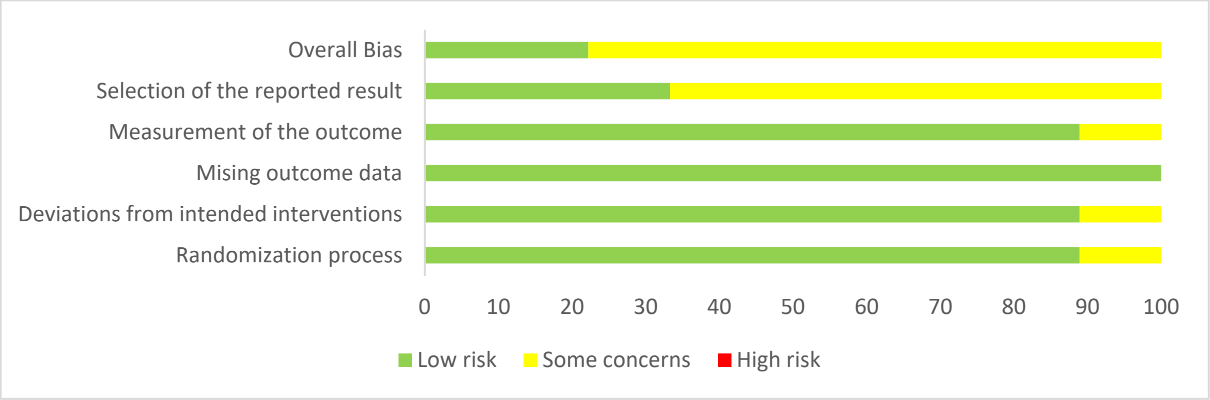

Figure S8: Risk of bias assessment on study level (A) and across studies (B) for studies reporting length of hospital stay.

Figures S9 RoB—Reoperation and readmission rate.

A

+ Low risk
? Some concerns
− High risk

	Randomization process	Deviations from intended interventions	Missing outcome data	Measurement of the outcome	Selection of the reported result	**Overall**
Gomez-Izquierdo, 2017	+	+	+	+	+	+
Joosten, 2018	+	+	+	+	+	+

B

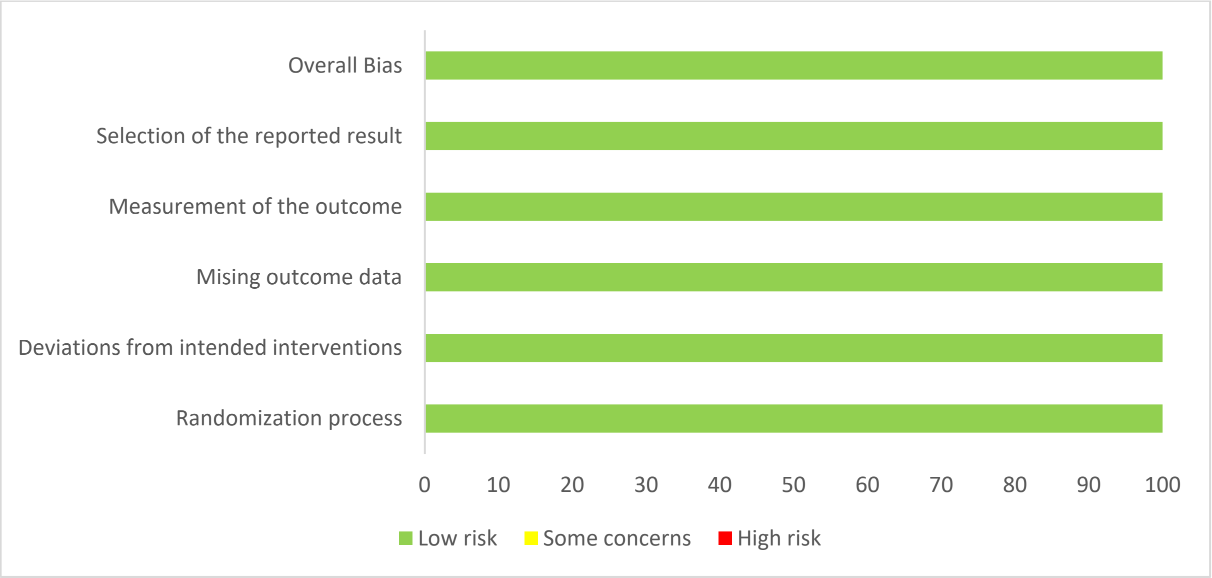

Figure S9: Risk of bias assessment on study level (A) and across studies (B) for studies reporting readmission rate.

Figures S10: RoB—Overall complication.

A

+ Low risk
? Some concerns
– High risk

	Randomization process	Deviations from intended interventions	Missing outcome data	Measurement of the outcome	Selection of the reported result	**Overall**
Gomez-Izquierdo, 2017	+	+	+	+	+	+
Brandstrup, 2012	+	+	+	+	?	?
Calvo-Vecino, 2018	+	+	+	+	?	?
Joosten, 2018	+	+	+	+	+	+
Ratti, 2016	+	+	+	+	?	?
Wen, 2016	?	?	+	?	?	?
Tang, 2021	+	+	+	?	?	?

B

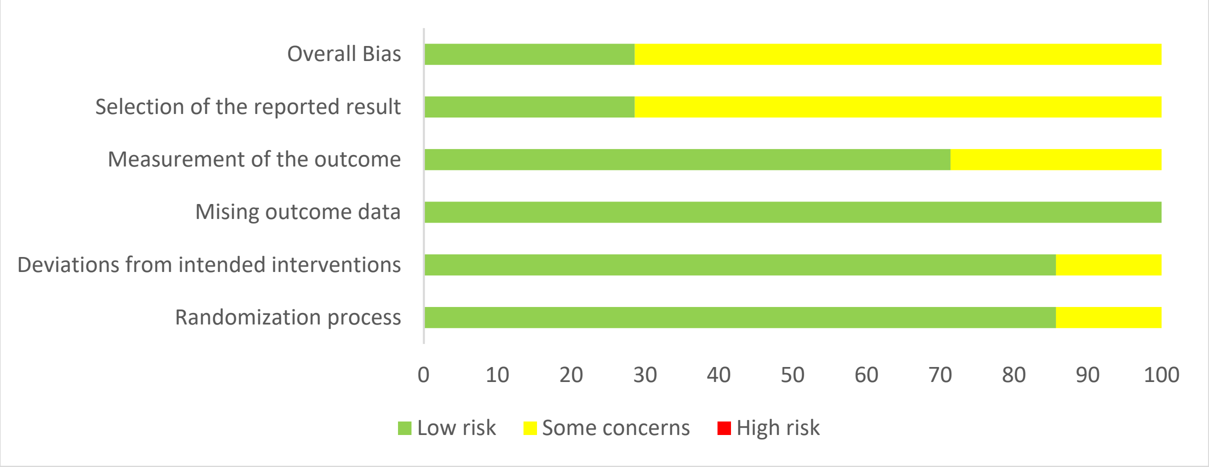

Figure S10: Risk of bias assessment on study level (A) and across studies (B) for studies reporting overall complications.

Figure S11: RoB—Time to first flatus.

A

+ Low risk
? Some concerns
– High risk

	Randomization process	Deviations from intended interventions	Missing outcome data	Measurement of the outcome	Selection of the reported result	**Overall**
Wen, 2016	?	+	+	?	?	**?**
Joosten, 2019	+	+	+	+	+	**+**
Gomez-Izquierdo, 2017	+	+	+	+	+	**+**
Li, 2021	+	+	+	+	?	**?**
Tang, 2021	+	+	+	+	?	**?**

B

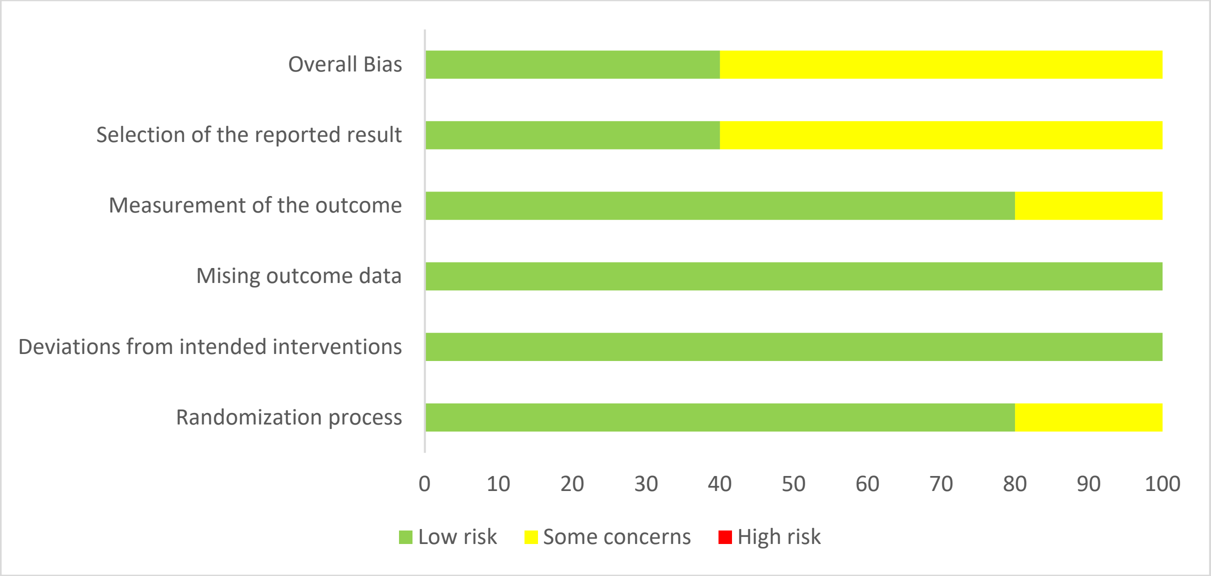

Figure S11: Risk of bias assessment on study level (A) and across studies (B) for studies reporting time to first flatus.

Figure S12: RoB—Time to first stool.

A

+ Low risk
? Some concerns
– High risk

	Randomization process	Deviations from intended interventions	Missing outcome data	Measurement of the outcome	Selection of the reported result	**Overall**
Wen, 2016	?	?	+	?	?	**?**
Joosten, 2018	+	+	+	+	+	**+**
Yin, 2018	+	+	+	?	+	**?**
Mei, 2018	?	?	+	?	?	**?**
Liu, 2019	+	+	+	+	?	**?**

B

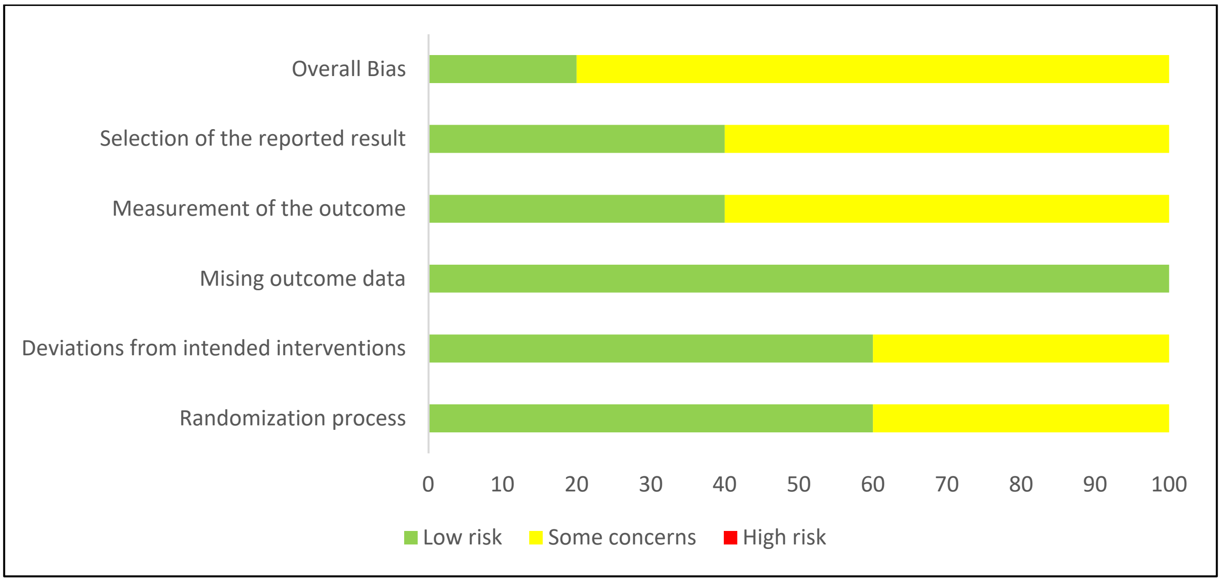

Figure S12: Risk of bias assessment on study level (A) and across studies (B) for studies reporting time to first stool.

Figure S13: RoB—Intraoperative fluid requirement.

A

+ Low risk
? Some concerns
– High risk

	Randomization process	Deviations from intended interventions	Missing outcome data	Measurement of the outcome	Selection of the reported result	**Overall**
Wen, 2016	?	+	+	+	?	**?**
Gomez-Izquierdo, 2017	+	+	+	+	+	**+**
Yin, 2018	+	+	+	+	?	**?**
Joosten, 2018	+	+	+	+	+	**+**
Liu, 2019	+	+	+	+	?	**?**
Cho, 2021	+	+	+	+	?	**?**
Tang, 2021	+	+	+	+	?	**?**
Mühlbacher, 2021	+	+	+	+	?	**?**

B

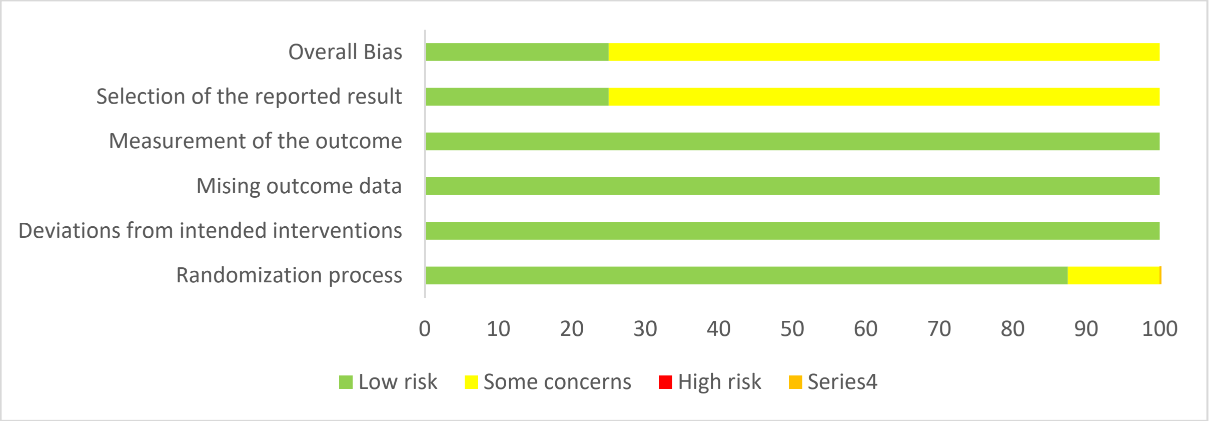

Figure S13: Risk of bias assessment on study level (A) and across studies (B) for studies reporting intraoperative fluid requirement.

Figure S14: RoB—Intraoperative vasopressor requirement.

A

+ Low risk
? Some concerns
– High risk

	Randomization process	Deviations from intended interventions	Missing outcome data	Measurement of the outcome	Selection of the reported result	**Overall**
Wen, 2016	?	+	+	+	?	**?**
Mei, 2018	?	+	+	+	?	**?**
Joosten, 2018	+	+	+	+	+	**+**
Gomez-Izguierdo, 2017	+	+	+	+	+	**+**
Demirel, 2017	?	+	+	+	?	**?**
Yin, 2018	+	+	+	+	?	**?**
Mühlbacher, 2021	+	+	+	+	?	**?**
Tang, 2021	+	+	+	+	?	**?**
Cho, 2021	+	+	+	+	?	**?**

B

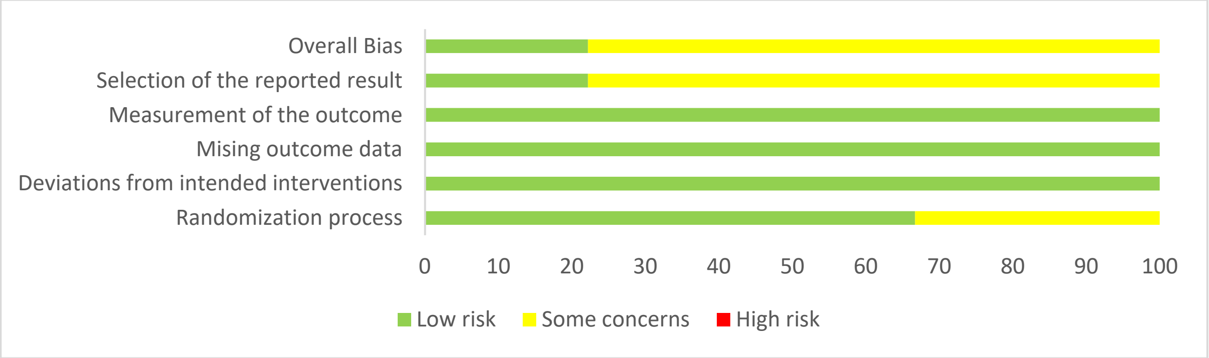

Figure S14: Risk of bias assessment on study level (A) and across studies (B) for studies reporting intraoperative vasopressor requirement.

Figure S15: RoB—Intraoperative urinary output.

A

+ Low risk
? Some concerns
– High risk

	Randomization process	Deviations from intended interventions	Missing outcome data	Measurement of the outcome	Selection of the reported result	**Overall**
Mei, 2018	?	+	+	+	?	**?**
Yin, 2018	+	+	+	+	?	**?**
Joosten, 2018	+	+	+	+	+	**+**
Liu, 2019	+	+	+	+	?	**?**
Li, 2021	+	+	+	+	?	**?**

B

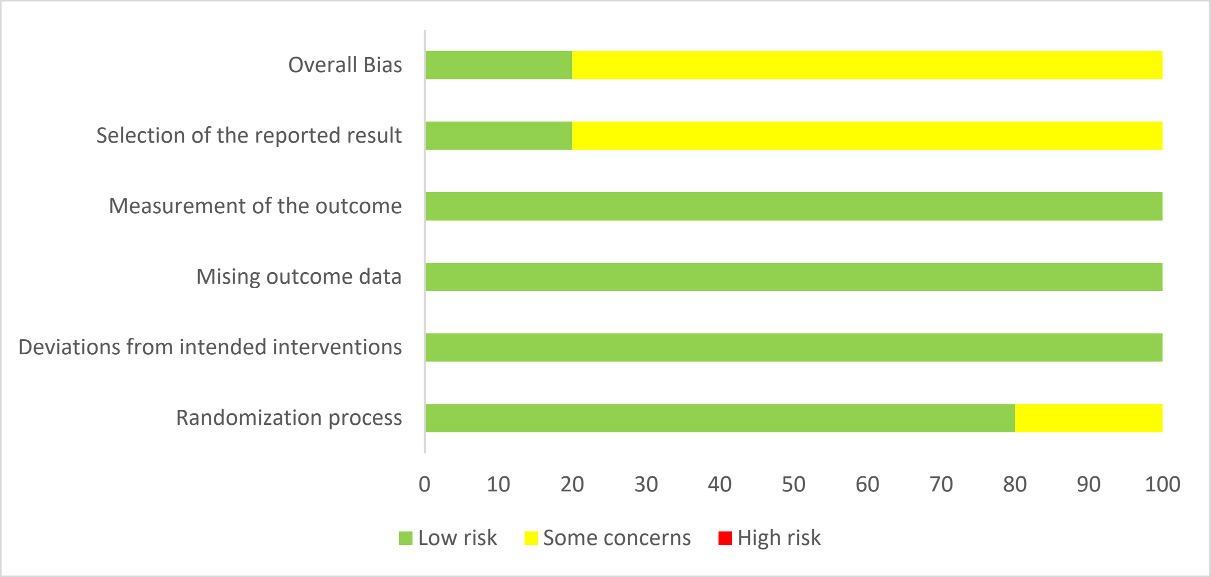

Figure S15: Risk of bias assessment on study level (A) and across studies (B) for studies reporting intraoperative urinary output.

Figure S16: RoB—Serum lactate levels at the end of the operation.

A

+ Low risk
? Some concerns
– High risk

	Randomization process	Deviations from intended interventions	Missing outcome data	Measurement of the outcome	Selection of the reported result	**Overall**
Mei, 2018	?	+	+	?	?	**?**
Yin, 2018	+	+	+	+	?	**?**
Liu, 2019	+	+	+	+	?	**?**

B

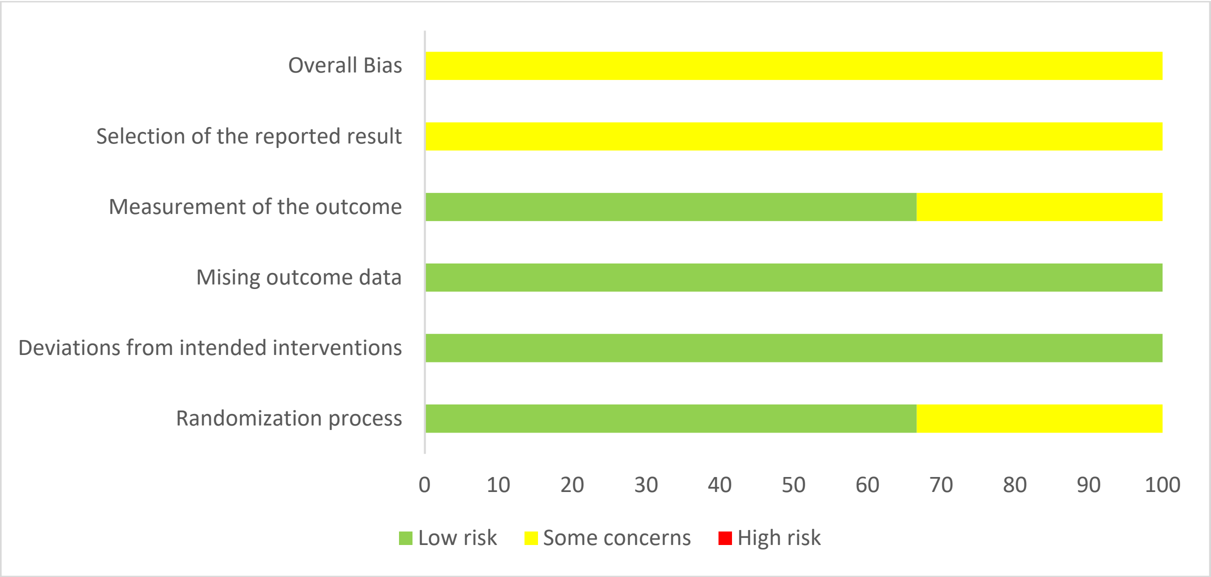

Figure S16: Risk of bias assessment on study level (A) and across studies (B) for studies reporting serum lactate levels at the end of the operations.

Supplementary Table S4: Certainty assessment.

Certainty Assessment						
**Outcomes**	**Risk of Bias**	**Inconsistency**	**Indirectness**	**Imprecision**	**Publication Bias**	**Overall Certainty of Evidence**
**Length of hospital stay (days)**	serious [a]	serious [c]	serious [e]	serious [f]	none	⊕◯◯◯ VERY LOW
**Time to first stool after the operation (hours)**	serious [b]	not serious	serious [e]	not serious	none	⊕⊕◯◯ LOW
**Time to first flatus after the operation (hours)**	serious [a]	serious [c]	serious [e]	serious [f]	none	⊕◯◯◯ VERY LOW
**Intraoperative fluid requirement (mL)**	serious [a]	serious [c]	serious [e]	serious [f]	none	⊕◯◯◯ VERY LOW
**Intraoperative vasopressor requirement**	serious [a]	serious [d]	serious [e]	serious [f]	none	⊕◯◯◯ VERY LOW
**Intraoperative urinary output standardized for length of surgery (mL/h)**	serious [a]	not serious	serious [e]	serious [f]	none	⊕◯◯◯ VERY LOW
**Serum lactate levels at the end of the operation (mmol/L)**	serious [a]	not serious	serious [e]	not serious	none	⊕⊕◯◯ LOW

Supplementary Table S4: Certainty assessment.

a. Probable high risk of bias due to the high proportion of the selective reporting.
b. Probable high risk of bias due to the high proportion of selective reporting and the chance of the inadequate measurement of the outcome.
c. Serious inconsistency due to high heterogeneity.
d. Serious inconsistency due to the opposite effects of three out of eight studies, which results in high variation.
e. Serious indirectness. Intraoperative fluid protocols of the control group diverged.
f. Serious imprecision due to the optimal sample size not being reached.

Figure S17: Leave-one-out analysis—Length of hospital stay.

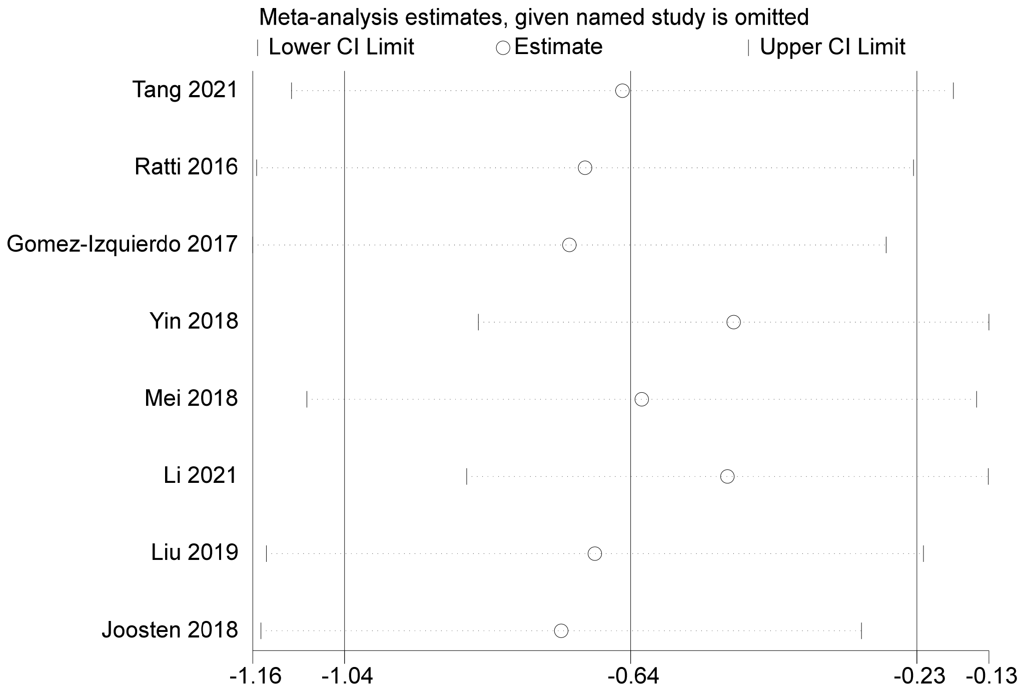

Figure S17: Leave-one-out sensitivity analysis for studies reporting length of hospital stay. Each row shows overall WMD and CI with the omission of the indicated study. There is no study of which its omission would change the statistical significance.

Figure S18: Leave-one-out analysis—Time to first stool.

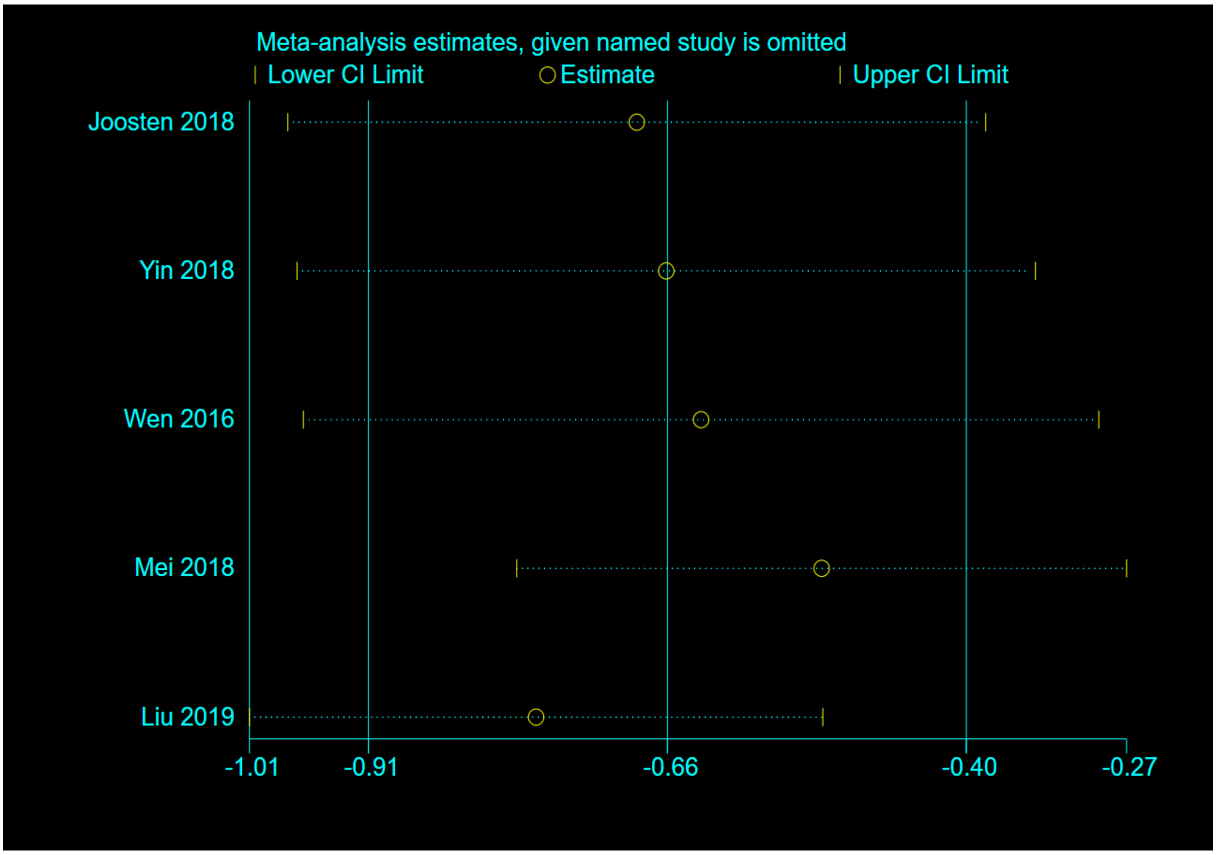

Figure S18: Leave-one-out sensitivity analysis for studies reporting time to first stool. Each row shows overall WMD and CI with the omission of the indicated study. There is no study of which its omission would change the statistical significance.

Figure S19: Leave-one-out analysis—Time to first flatus.

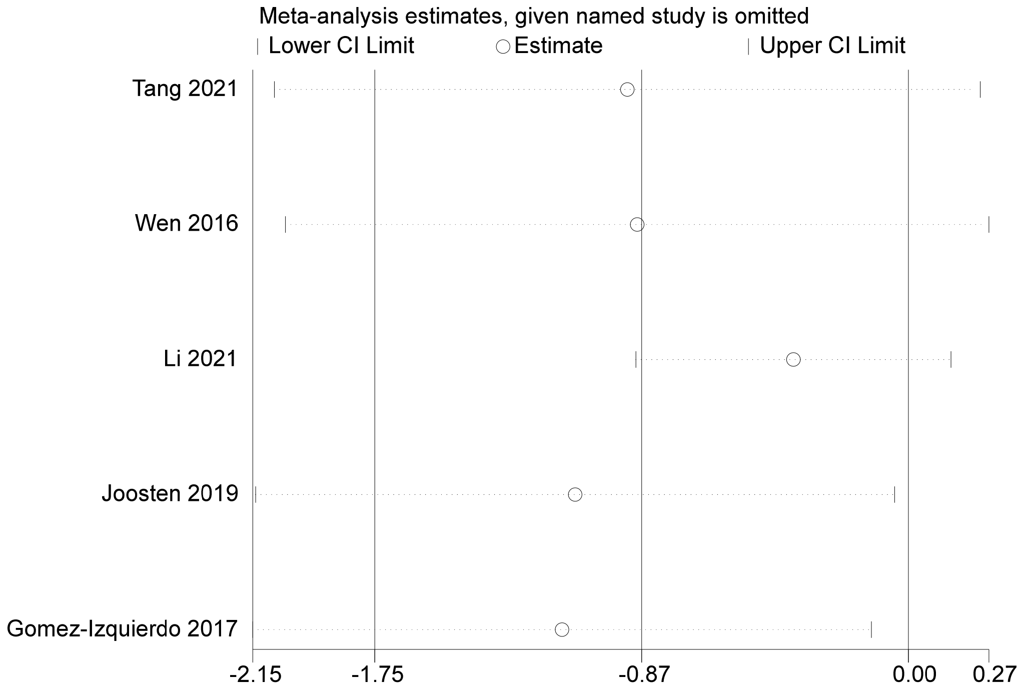

Figure S19: Leave-one-out sensitivity analysis for studies reporting time to first flatus. Each row shows overall WMD and CI with the omission of the indicated study. Omission of the studies of Tang et al., Wen et al., and Li et al. would change the statistical significance.

Figure S20: Leave-one-out analysis—Intraoperative fluid requirement.

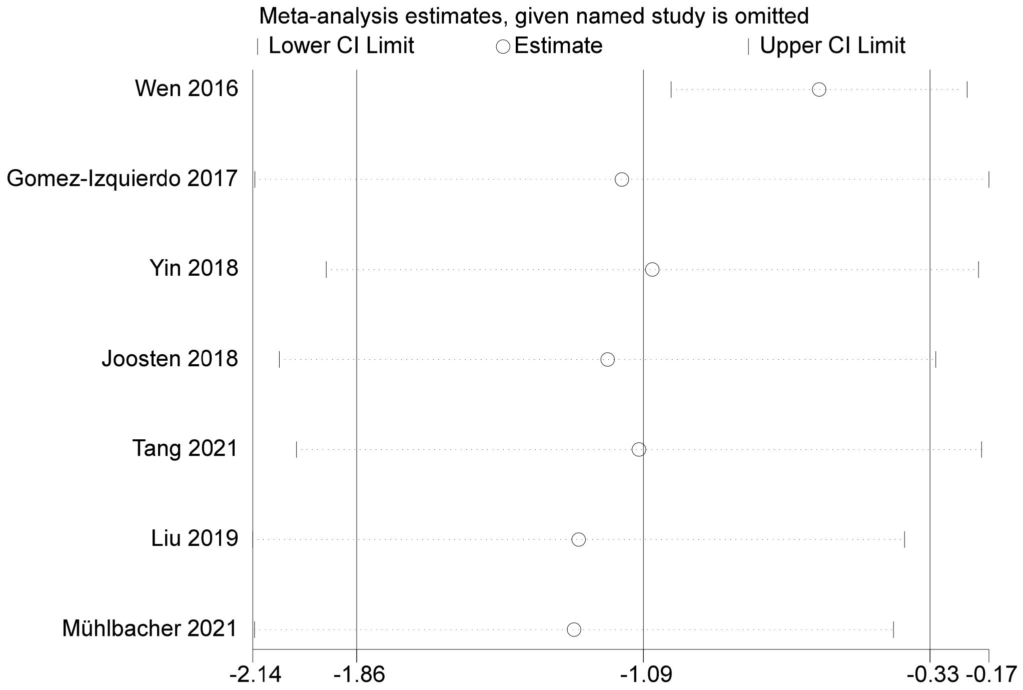

Figure S20: Leave-one-out sensitivity analysis for studies reporting intraoperative fluid requirement. Each row shows overall WMD and CI with the omission of the indicated study. There is no study of which its omission would change the statistical significance.

Figure S21: Leave-one-out analysis—Intraoperative vasopressor requirement.

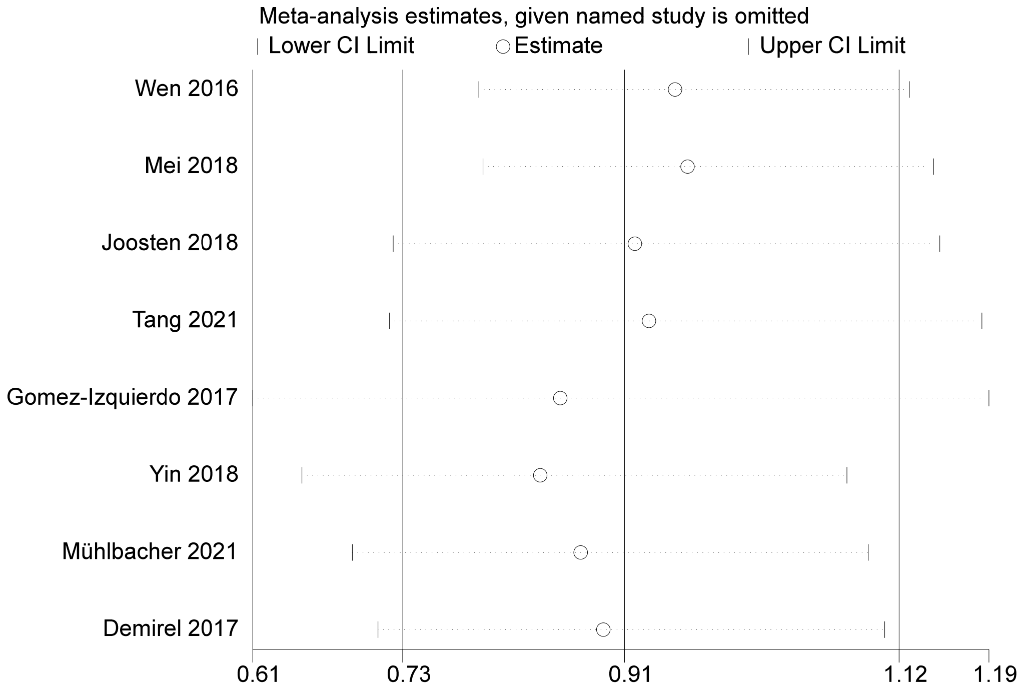

Figure S21: Leave-one-out sensitivity analysis for studies reporting intraoperative vasopressor requirement. Each row shows overall RR and CI with the omission of the indicated study. There is no study of which the omission would change the statistical significance.

Figure S22: Leave-one-out analysis—Intraoperative urinary output.

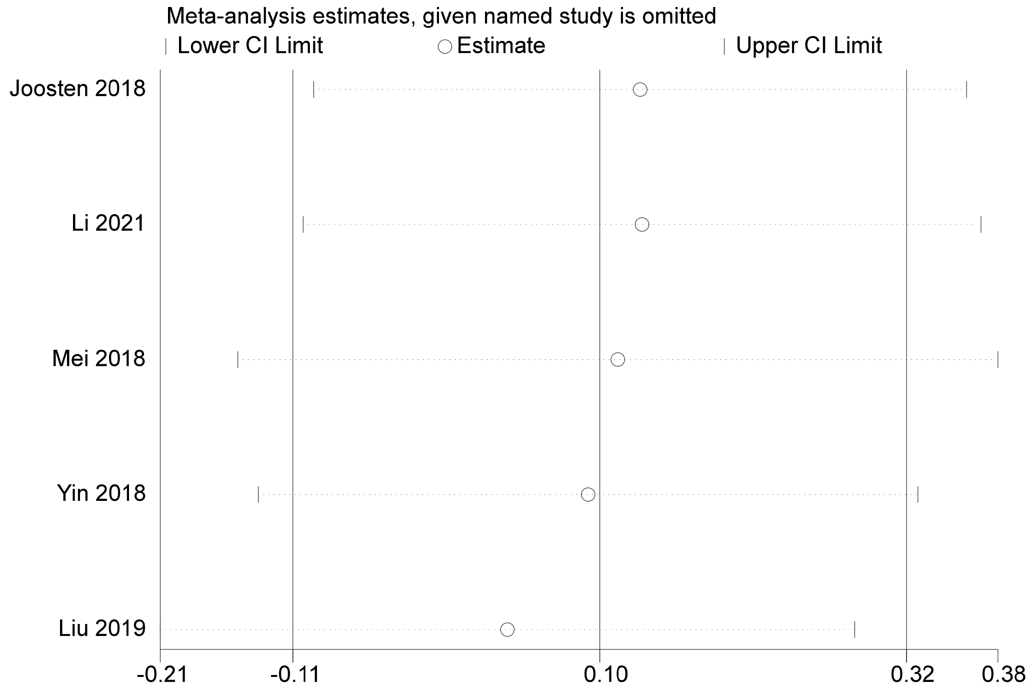

Figure S22: Leave-one-out sensitivity analysis for studies reporting intraoperative fluid requirement. Each row shows overall SMD and CI with the omission of the indicated study. There is no study of which its omission would change the statistical significance.
